# Supplementary material for: The Decline and Fall of the Current Chemotherapy Paradigm in Soft Tissue Sarcoma
Source: Cancers (Basel). 2025 Apr 1;17(7):1203. doi: 10.3390/cancers17071203 (PMC11987756; doi:10.3390/cancers17071203)
Supplement: Supplementary file 1 [file cancers-17-01203-s001.zip › cancers-3514103-supplementary.pdf]

Table S1. Randomized Clinical Trials Involving Anthracyclines in Metastatic Soft Tissue Sarcoma.

Study	Sarcoma Subtype	Intervention	Control	Number of Patients Evaluated Intervention	Number of Patients Evaluated Control	Median PFS Intervention	Median PFS Control	Median OS Intervention	Median OS Control	Summary of results
Schoenfeld et al. 1982	All (majority STS)	VAC, VAdrC	Dox	VAC: 64, VAdrC: 70	66	VAC: 9 weeks, VAdrC: 15 weeks	17 weeks	VAC: 41 weeks, VAdrC 34 Weeks	37 Weeks	PFS improved with Dox (p<0.03), no statistical difference in OS
Omura et al. 1983	Uterine sarcoma	Dox/DITC	Dox	106	120	NR	NR	7.3 months	7.7 months	No statistical difference in OS
Mouridsen et al. 1987	STS	Epirubicin	Dox	84	83	12 weeks	15 weeks	48 weeks	41 weeks	No statistical difference in PFS or OS
Borden et al. 1987	STS	Dox/DITC, LD weekly Dox	Dox	Dox/DITC: 92, LD weekly Dox: 89	94	Dox/DITC: 3.6 months, LD weekly Dox: 2.4 months	3.0 months	Dox/DITC: 8.0 months, LD Dox: 8.4 months	8.0 months	No statistical difference in PFS or OS
Antman et al. 1993	All (majority STS)	MAID	Dox/DITC	170	170	6.1 months	3.9 months	11.9 months	13.3 months	PFS improved with MAID (p=0.02), OS improved with Dox/DITC likely due to toxicity of MAID (p=0.04)
Santoro et al. 1995	STS	CYVADIC, Dox/Ifos	Dox	CYVADIC: 142, Dox/Ifos: 258	263	NR	NR	CYVADIC: 51 weeks, Dox/Ifos: 55 weeks	52 weeks	No statistical difference in PFS or OS
Nielsen et al. 1998	STS	HD Epirubicin (1 day vs 3 day)	Dox	1D E: 111, 3D E 111	112	NR	NR	1 day epirubicin: 47 weeks, 3 day epirubicin: 45 weeks	45 weeks	No statistical difference in PFS at one year or OS
Verweij et al. 2000	STS	Docetaxel	Dox	43	43	7 weeks	24 weeks	42 weeks	53 weeks	PFS improved with Dox (p=0.14), no statistical difference in OS
Judson et al. 2001	STS	Liposomal Dox	Dox	50	45	65 days	82 days	320 days	246 days	No statistical difference in PFS or OS
Lorigan et al. 2007	STS	Ifos bolus and Ifos infusional	Dox	Ifos Bolus: 105, Ifos infusional: 102	110	Ifos Bolus: 2.16 months, Ifos infusional: 3.0 months	2.52 months	Ifos Bolus: 10.92 months, Ifos infusional: 10.92 months	12.00 months	No statistical difference in PFS or OS
Maurel et al. 2009	STS	Dox/Ifos	Dox	65	67	24 weeks	26 weeks	NR	NR	No statistical difference in PFS or OS
Demetri et al. 2012	STS	Dox/conatumumab	Dox	86	42	5.6 months	6.4 months	18 months	21.6 months	No statistical difference in PFS or OS
Gelderblom et al. 2014	STS	Brostallicin	Dox	79	39	0.13 years	0.51 years	1.06 years	1.10 years	PFS improved with Dox (p value not in manuscript), no statistical difference in OS
Judson et al. 2014	STS	Dox/Ifos	Dox	227	228	7.4 months	4.6 months	14.3 months	12.8 months	PFS improved with combination (p=0.003), no statistical difference in OS
Bui-Nguyen et al. 2015	STS	Trabectedin 3H, Trabectedin 24H	Dox	Trabectedin 3H: 47, Trabectedin 24H: 43	43	Trabectedin 3H: 2.8 months, Trabectedin 24H: 3.1 months	5.5 months	NR	NR	No statistical difference in PFS, median OS not reached

Chawla et al. 2015	STS	Aldoxorubicin	Dox	83	40	5.6 months	2.7 months	15.8 months	14.3 months	PFS improved with alDox (p=0.02), no statistical difference in OS
Ryan et al. 2016	STS	Dox/Palifosfamide	Dox	226	221	6.0 months	5.2 months	15.9 months	16.9 months	No statistical difference in PFS or OS
Martin-Broto et al. 2016	STS	Dox and trabectedin	Dox	54	59	5.7 months	5.5 months	13.3 months	13.7 months	No statistical difference in PFS or OS
Tap et al. 2016	STS	Dox/olaratumumab	Dox	66	67	6.6 months	4.1 months	26.5 months	14.7 months	PFS improved with combination (stratified p=0.0615), OS improved with combination (stratified p=0.0003)
Seddon et al. 2017	STS	Gemcitabine/docetaxel	Dox	126	128	23.7 weeks	23.3 weeks	67.3 weeks	76.3 weeks	No statistical difference in PFS or OS
Tap et al. 2020	STS	Dox/olaratumumab	Dox	258	251	5.4 months	6.8 months	20.4 months	19.7 months	No statistical difference in PFS or OS
Tap et al. 2020	STS	Dox/evofosfamide	Dox	317	323	6.3 months	6.0 months	29.2 months	28.3 months	No statistical difference in PFS or OS
Grünwald et al. 2020	STS	Pazopanib	Dox	81	39	4.4 months	5.3 months	12.3 months	14.3 months	No statistical difference in PFS or OS
Pautier et al. 2024	LMS	Dox/Trabectedin	Dox	74	76	12 months	6 months	33 months	24 months	PFS improved with combination (p<0.0001), OS improved with combination (p value not documented)

STS, Soft tissue sarcoma; Dox, doxorubicin; DITC, dacarbazine; Ifos, ifosfamide; VAC, vincristine/dactinomycin/cyclophosphamide; VAdriC vincristine/doxorubicin/cyclophosphamide; PFS, progression free survival; OS overall survival
